# Supplementary material for: Photodynamic Therapy with Protoporphyrin IX Precursors Using Artificial Daylight Improves Skin Antisepsis for Orthopedic Surgeries
Source: Microorganisms. 2025 Jan 18;13(1):204. doi: 10.3390/microorganisms13010204 (PMC11767567; doi:10.3390/microorganisms13010204)
Supplement: Supplementary file 1 [file microorganisms-13-00204-s001.zip › microorganisms-3358434-supplementary.pdf]

Supplementary Material

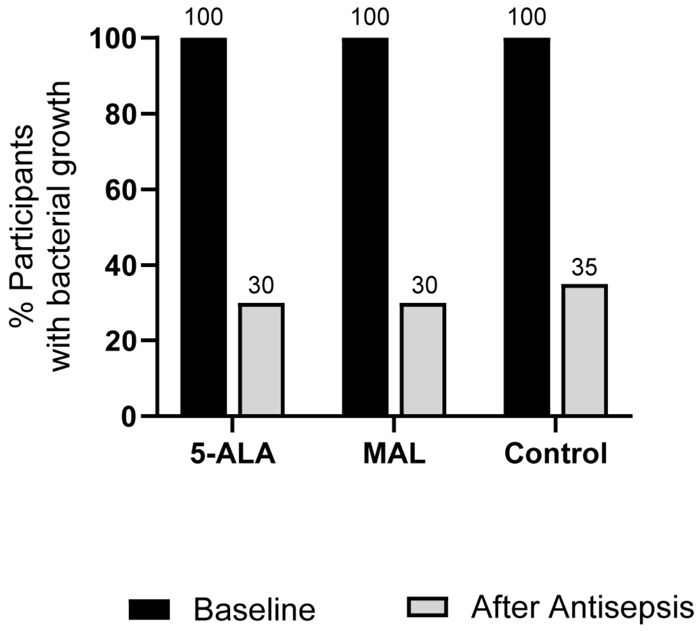

Supplementary Figure S1. **Long lasting effect of PDT-DL on bacterial growth.** Percentage of participants with positive bacterial three days post treatment (day 4) before and after skin antisepsis. Numbers above the bars indicate % Participants with bacterial growth. Control only contains n = 20 participants instead of n = 30. See methods for further information. PDT-DL, Photodynamic therapy with artificial daylight; 5-ALA, 5-Aminolevulinic acid; MAL, Methyl aminolevulinate.

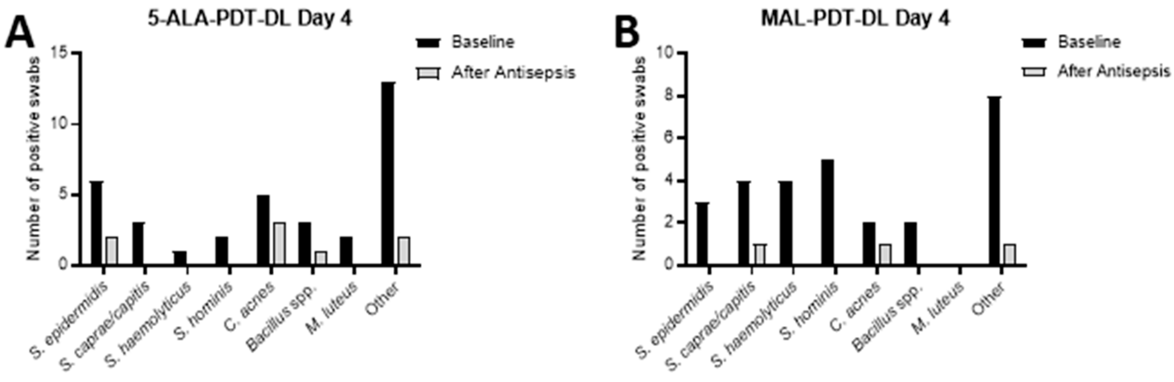

Supplementary Figure S2. Long-lasting effect of PDT-DL on specific bacterial species. Number of positive samples on day 4 for **(A)** the 5-ALA-PDT-DL arm and **(B)** the MAL-PDT-DL arm at baseline (blue) and after antisepsis (turquoise). Other included *Corynebacterium* spp., *Staphylococcus warneri/pasteuri, Staphylococcus* spp., *Moraxella osloensis, Cutibacterium avidum, Staphylococcus. cohnii, Kocuria* spp., *Dermabacter* spp. *Facklamia hominis, Staphylococcus lugdunensis, Pseudomonas* spp., Gram-positive rods, *Bacillus subtillis, Bacillus pumilus, Enterococcus faecium, Staphylococcus saprophyticus, Enterococcus faecalis, Bacillus megaterium, Staphylococcus. aureus, Cutibacterium* spp. and *Enterococcus gallinarum*.
